# Suicide in Schizophrenia: An Educational Overview

**DOI:** 10.3390/medicina55070361

**Published:** 2019-07-10

**Authors:** Leo Sher, René S. Kahn

**Affiliations:** 1 James J. Peters Veterans’ Administration Medical Center, Bronx, New York, NY 10468, USA; 2 Icahn School of Medicine at Mount Sinai, New York, NY 10029, USA

**Keywords:** suicide, schizophrenia, antipsychotics, depression

## Abstract

Suicide is an important public health problem. The most frequent psychiatric illnesses associated with suicide or severe suicide attempt are mood and psychotic disorders. The purpose of this paper is to provide an educational overview of suicidal behavior in individuals with schizophrenia. A lifetime suicide rate in individuals with schizophrenia is approximately 10%. Suicide is the largest contributor to the decreased life expectancy in individuals with schizophrenia. Demographic and psychosocial factors that increase a risk of suicide in individuals with schizophrenia include younger age, being male, being unmarried, living alone, being unemployed, being intelligent, being well-educated, good premorbid adjustment or functioning, having high personal expectations and hopes, having an understanding that life’s expectations and hopes are not likely to be met, having had recent (i.e., within past 3 months) life events, having poor work functioning, and having access to lethal means, such as firearms. Throughout the first decade of their disorder, patients with schizophrenia are at substantially elevated suicide risk, although they continue to be at elevated suicide risk during their lives with times of worsening or improvement. Having awareness of symptoms, especially, awareness of delusions, anhedonia, asociality, and blunted affect, having a negative feeling about, or non-adherence with, treatment are associated with greater suicide risk in patients with schizophrenia. Comorbid depression and a history of suicidal behavior are important contributors to suicide risk in patients with schizophrenia. The only reliable protective factor for suicide in patients with schizophrenia is provision of and compliance with comprehensive treatment. Prevention of suicidal behavior in schizophrenia should include recognizing patients at risk, delivering the best possible therapy for psychotic symptoms, and managing comorbid depression and substance misuse.

## 1. Suicide as a Medical and Social Problem

Suicide is an important public health problem [1,2]. According to the World health Organization (WHO), each year, about one million people die by suicide across the world [1]. It implies that every 40 s, an individual dies by suicide someplace on the globe and many more people make non-lethal suicide attempts. It has been proposed that the number of persons who make non-lethal suicide attempts is about 10–15 times the amount of people who die by suicide. Deaths by suicide and non-lethal suicide attempts greatly affect families, communities, and societies. In the United States, the expenses of managing suicide attempters and examining deaths by suicide have been assessed as being $190 million per year [3]. A recent study showed that non-lethal suicide attempts are associated with decreased life span [4]. It is worth noting that this study found that most additional deaths are attributable to physical/medical conditions.

Rates of suicide death are very significant in many countries across the world [1]. A report issued in November 2018 by the United States Centers for Disease Control and Prevention indicates that from 1999 through 2017, the age-adjusted rate of suicide in the United States rose 33% from 10.5 to 14.0 per 100,000 [5]. It is possible that suicide rates are underestimated. A lot of suicide deaths may be wrongly recorded as ‘unnatural’ or ‘undetermined’ deaths. Actual suicide rates may well be 10%–50% higher than reported.

Globally, men die by suicide 3–7 times more often than women [1,6]. The sex dissimilarities in suicide rates are particularly substantial in Eastern European nations [1,6]. In the United States, in 2017, the age-adjusted suicide rate for men (22.4 per 100,000) was 3.67 times larger than for women (6.1 per 100,000) [5].

Studies in the U.S. suggest that more than 90% of victims of suicide have a psychiatric disorder [2,7,8,9]. Furthermore, most people who attempt suicide have a psychiatric disorder. The most frequent psychiatric illnesses associated with suicide or severe suicide attempt are mood and psychotic disorders [2,7,8,9,10]. A 5-year follow-up study of 1065 patients with psychotic disorders conducted by the World Health Organization (WHO) found that “the risk for suicide in schizophrenia is as great, if not greater, than the risk of suicide associated with affective disorders” [11]. Alcohol and drug abuse, anxiety and personality disorders are also associated with an elevated suicide risk [12,13,14,15].

Medical disorders, particularly illnesses associated with chronic pain, significantly increase suicide risk [16,17,18,19,20]. While many deaths due to opioid overdoses are accidental, an increasing amount of data indicates that the presence of pain plays a role in the decision to end life via opioid overdoses [19]. Neurological conditions such as stroke, epilepsy, head injury, or Huntington’s disease also confer greater suicide risk [20,21]. Obstacles to preventing suicidal behavior include inadequate rates of detection of persons with psychiatric illnesses, insufficient dissemination of evidence-based methods among community providers, and enormous complexity in detecting imminent suicide risk, even in persons who are being cared for psychiatric conditions [22,23].

Adolescence is a period of transition from childhood to adulthood and also, a time of increased vulnerability to psychiatric disorders including psychotic disorders [24]. A systematic review suggests that the number of adversities or negative life events experienced by adolescents appear to have a positive dose–response relationship with youth suicidal behavior [25]. Hence, traumatic experiences during adolescence may contribute to the pathophysiology of both psychotic disorders and suicidal behavior.

The purpose of this paper is to provide an educational overview of suicidal behavior in individuals with schizophrenia. Most literature searches were performed using the PubMed database.

## 2. Epidemiology of Suicidal Behavior in Schizophrenia

As early as 1911, E. Bleuler characterized “the suicidal drive” as the “most serious of all schizophrenic symptoms” [26]. In 1919, Kraepelin stated that suicide happened in both acute and chronic stages of schizophrenia [27]. In 1939, before contemporary treatments became available, Rennie [28] observed that 11 percent of 500 patients with schizophrenia had died by suicide throughout a 20-year follow-up period.

Contemporary research studies indicate that a lifetime rate of suicide in individuals with schizophrenia is between 4% and 13%, while the modal rate is about 10% [29]. The reported rates of suicide attempts in patients with schizophrenia vary between from 18% to 55% [29,30,31].

Considerable evidence suggests that schizophrenia decreases the longevity by about 10 years [32]. Suicide is the largest contributor to the decreased life expectancy in individuals with schizophrenia. Recognition of suicide risk factors in patients with schizophrenia is vital in order to enhance patient treatment and advance approaches to decrease the incidence of suicide in patients with schizophrenia.

## 3. Demographic and Psychosocial Risk Factors

Studies and observations suggest that the following demographic and psychosocial factors increase the risk of suicide in individuals with schizophrenia [10,29,33,34,35,36]:

• Younger age
• Being male
• Being unmarried
• Living alone
• Being unemployed
• Being intelligent
• Being well-educated
• Good premorbid adjustment or functioning
• Having high personal expectations and hopes
• Having an understanding that life’s expectations and hopes are not likely to be met
• Having had recent (i.e., within past 3 months) life events
• Having poor work functioning
• Having access to lethal means, such as firearms

Studies have demonstrated that suicidal behavior takes place when patients with schizophrenia are younger than age 45 years [37,38,39]. However, this association is probably more related to the onset of schizophrenia than to age itself.

Similar to the general population and patients with other psychiatric disorders, males with schizophrenia die by suicide more frequently than females with schizophrenia, but the gender difference among suicide victims with schizophrenia is considerably less (60% vs. 40%) [40,41,42]. Unlike the general population in which females usually make more non-lethal suicide acts than do men, rates of suicide attempts among individuals with schizophrenia have not been found to differ by gender [42,43,44].

Being single and being unemployed are suicide risk factors for individuals with schizophrenia [29,33,34,42,45]. Some researchers believe that there is a problem with interpreting these findings because most persons with schizophrenia are single and unemployed.

## 4. Risk Factors Related to Symptomatology and the Course of Illness

Throughout the first decade of their disorder, patients with schizophrenia are at substantially elevated suicide risk, although they continue to be at elevated suicide risk during their lives, with times of worsening or improvement [33,42,46]. Excess mortality due to suicide during the 40-year follow up of 200 patients with schizophrenia was as follows: 44 percent of the patients with schizophrenia who died by suicide committed suicide during the first decade of observation, 22 percent during the second decade, and another 22 percent during the third decade [47].

Studies indicate that suicide risk is significantly elevated during the first psychotic break [29,46,48,49,50]. Research of first-episode patients usually has higher estimations of suicide rates than studies with lengthier follow up periods. It has been noted that during early stages of schizophrenia, limited suicidal ideation may quickly intensify to a suicide attempt. A delay in getting psychiatric treatment may substantially contribute to elevated suicide risk early in the course of schizophrenia. Other suicide risk factors early in the course of schizophrenia include earlier age of the onset of psychotic symptoms, female sex, suicidal plans, a history of suicide attempt, seriousness of psychiatric pathology, a history of emotional trauma, and decent insight. Suicidality during the first psychotic episode is associated with a good understanding of the situation and beliefs about bad outcomes for psychotic conditions.

Having had an earlier age of onset, being in an early part of the course of illness, having awareness of symptoms, especially, awareness of delusions, anhedonia, asociality, and blunted affect, having a negative feeling about, or non-adherence with, treatment are associated with greater suicide risk in patients with schizophrenia [33,51,52,53]. In addition, a number of psychiatric admissions is an important suicide risk factor in both inpatients and outpatients with schizophrenia [35,51].

Recurrent relapses, a significant severity of the disease, a descending shift in societal and occupational functioning, and a true and realistic understanding of the harmful influence of the disorder are regarded as schizophrenia-specific suicide risk factors [10]. Patients with the paranoid subtype of schizophrenia are eight times more likely to die by suicide in comparison with patients with the deficit subtype of schizophrenia [54]. Delusions are associated with more suicidal behavior in individuals with schizophrenia [53]. A research report indicates that hostility at hospital admission is linked with long-term suicidal risk [54].

Lack of adherence to antipsychotic drugs may increase suicide risk [53,55]. For example, a large follow-up study of patients released from hospitals after a hospitalization related to the first episode of schizophrenia showed that not getting antipsychotic drugs led to a 12-fold rise in the relative risk of all-cause mortality and a 37-fold rise in suicide mortality [55].

It is important to note that as a result of psychotic disorganization, an individual with schizophrenia may be involved in very unsafe, risky behavior without understanding otherwise predictable threats [29]. Such behavior may look like suicidal behavior.

## 5. Risk Factors Related to Comorbid Disorders

Comorbid depression and a history of suicidal behavior are important contributors to suicide risk in patients with schizophrenia [33,53,56]. One study showed that depressive symptoms, suicidal ideation and plans and a history of suicide attempts are amongst the most important forecasters of suicidal behavior in the early phases of schizophrenia [57]. Two studies suggest that patients with schizophrenia who were hospitalized after a suicide attempt had the greatest risk, of all variables examined, of dying by suicide [58,59]. Other works also indicate that suicide risk in individuals with schizophrenia is associated with mood syndromes, especially depressed mood, hopelessness and demoralization [29,33,53,60]. Panic symptoms may also contribute to suicidal behavior in patients with schizophrenia [61].

A systematic review indicates that four investigations recognized alcohol abuse as a predisposing aspect to suicidal behavior amongst individuals with schizophrenia, while three investigations recognized substance use disorders and one research investigation recognized smoking only [53]. However, one study has shown that neither alcohol nor drug abuse elevates suicide risk in individuals with schizophrenia [62]. Furthermore, a research group observed that abuse of stimulants, such as cocaine or amphetamine increases the risk of suicidal behavior in schizophrenia [63]. As noted above, the presence of medical and/or neurological disorders may increase suicide risk [16,17,18,19,20,21].

## 6. Risk Factors Related to Antipsychotic Medications

Some observations suggest that the side effects of antipsychotic medications may contribute to suicidality in individuals with schizophrenia [64,65,66]. It has been suggested that antipsychotics-induced akathisia, akinesia, tardive dyskinesia and depressogenic effects of antipsychotic medications may increase suicide risk. For example, it has been observed that there is a significant association among akathisia and suicidality in first-episode psychosis [65,66].

It is important to note that one study observed a lower suicide risk among patients with extrapyramidal symptoms [67]. The authors believe that this finding could potentially reflect greater adherence to antipsychotic treatment, exposure to higher doses, or polypharmacy in this patient group.

## 7. Neurobiological Aspects of Suicidal Behavior in Schizophrenia

Some lines of evidence suggest that suicidal behavior in schizophrenia has a neurobiological basis [68,69,70,71,72,73]. Dexamethasone suppression test (DST) deviations have been observed in patients with schizophrenia who attempted suicide [68,69]. For example, it has been observed that dexamethasone non-suppression may be associated with a history of suicide attempts among medication-free persons with schizophrenia [68]. Hypothalamic-pituitary-adrenal axis (HPA) hyperactivity leading to glucocorticoid neurotoxicity may be the primary way through which tissue injury occurs in several parts of the brain, as observed in neuroimaging investigations of suicide attempters with schizophrenia [70].

Some studies found lower 5-hydroxy acetic acid (5-HIAAA) levels in the cerebrospinal fluid (CSF) of suicidal patients in comparison to the CSF levels of non-suicidal individuals with schizophrenia [71,74], while other studies did not detect this difference [75,76,77]. One research group found that a blunted prolactin response to a D-fenfluramine administration was associated with suicidal behavior in individuals with a history of schizophrenia [78]. A significant link was found between single nucleotide polymorphisms ADRA2B rs1018351 and SLC6A3 rs403636 and a history of suicidal behavior in schizophrenia patients [73].

## 8. Prevention of Suicide in Patients with Schizophrenia

Suicide prevention in patients with schizophrenia is a complex task. Clinicians need to be trained in how to identify patients who are at high suicide risk. Careful management of psychotic symptoms, comorbid depression and substance use disorders is necessary to prevent suicide in individuals with schizophrenia.

Efforts in suicide prevention should focus on enhancing compliance with medications. Studies suggest that antipsychotic medications, including clozapine, risperidone, olanzapine, and quetiapine may reduce suicide risk [33,79,80,81,82,83].

Several studies demonstrated efficacy of clozapine for the management of suicidality in schizophrenia [81,82]. It has been proposed that this decline in suicidal behavior can be ascribed to reduction in depressive symptomatology [84]. In December 2002, the U.S. Food and Drug Administration (FDA) granted indication for clozapine to decrease the risk of recurring suicidal behavior in individuals with schizophrenia/schizoaffective disorder [50]. Potentially, early use of clozapine may significantly reduce risk of suicide in patients with schizophrenia [53]. A recent study indicates that clozapine should be administered after patients with schizophrenia fail a single antipsychotic medication trial—not until two antipsychotic drugs have been attempted, as is the existing guideline [85]. Such an approach may reduce suicidality in individuals with schizophrenia.

Other second-generation antipsychotics may also have anti-suicidal properties [33,80,86]. For example, a retrospective study of the influences of atypical antipsychotic drugs on suicidal behavior in individuals with schizophrenia or schizoaffective disorder showed that among persons who made a suicide attempt, 16.1% took second-generation antipsychotic medications, whereas in the non-suicidal group 37% took second-generation antipsychotics [80]. Another study has shown a fourfold rise in suicide attempts among individuals with schizophrenia who stopped taking olanzapine or risperidone [86].

Long-acting injections are frequently used for the treatment of psychotic disorders [87]. Observations of the effect of depot antipsychotic medications on suicide risk have produced inconsistent results. For example, Battaglia et al. [87] showed that monthly intramuscular injections of fluphenazine decanoate reduce self-harm behavior in outpatients with histories of multiple suicide attempts. Shear et al. [88] reported suicides in two young men who developed severe akathisia after treatment with depot fluphenazine. A Cochrane review by Adams and Eisenbruch [89] compared depot fluphenazine medication to oral fluphenazine for treatment of schizophrenia and found no difference between fluphenazine hydrochloride and its depot form for outcomes such as depressed mood or suicide. A meta-analysis showed that pooled long-acting antipsychotics (aripiprazole, fluphenazine, haloperidol, olanzapine, paliperidone, risperidone, and zuclopenthixol) did not differ from pooled oral antipsychotics regarding all-cause death or death due to suicide [90]. Pompilli et al. [91] suggest that long-acting injections of second-generation antipsychotics can be an effective treatment strategy to improve adherence and may result in suicide prevention by targeting modifiable suicide risk factors.

Concurrent depression is a significant risk factor for suicidal behavior in individuals with schizophrenia [29,33,34]. Studies suggest a relation between a decrease in suicide risk and the use of antidepressants [92,93]. The use of antidepressants has been associated with a decrease in all-cause mortality when used together with antipsychotic drugs [94]. Psychiatrists should consider the addition of antidepressant medications for concurrent depression in patients with schizophrenia [29,33,34]. Furthermore, medications that reduce substance abuse (e.g., naltrexone or acamprosate) should be prescribed for patients with comorbid schizophrenia/schizoaffective disorder and substance use disorder.

Non-pharmacological approaches are also important in decreasing suicidal behavior in schizophrenia [33,34,95]. Psychosocial interventions play an important role in the treatment of suicidal patients with schizophrenia. It is vital to educate mental health providers about suicidology [34]. They need to be prepared to deal with the depression, anxiety, anguish, and hopelessness of suicidal individuals with schizophrenia. Empathic care and support are critical for decreasing suicidal risk [33,34,95]. Clinicians should recognize the patient’s distress, talk to the patient about his/her daily problems, and assist patients in establishing realistic goals.

Supportive, reality-orientated therapies are important in the management of patients with psychotic disorders [34]. Individual and group sessions can help patients to learn how to handle difficulties. Psychosocial interventions including cognitive-behavioral therapy, cognitive remediation, supportive therapy, supported education, training, and employment are important for successful management of schizophrenia [34,96]. One study found that cognitive therapy decreases suicidal ideation in individuals with schizophrenia [97]. Interventions such as vocational rehabilitation, social skill training, and supported employment may reduce social isolation and feeling of hopelessness, and consequently, decrease suicidality in schizophrenia patients.

Family interventions may reduce the risk of suicidal behavior, and therefore, should be a necessary component of a treatment plan of each patient with schizophrenia [98,99]. Such interventions considerably lessen rates of readmission and relapse in individuals with psychotic disorders and enhance their social and vocational performance [98,99]. Family interventions usually increase adherence to pharmacological therapy [99]. Relatives of patients with schizophrenia sometimes display excessive emotional reactions and convey intolerant, judgmental, and/or emotionally overinvolved attitudes toward patients [100,101,102]. Family members high in expressed emotion may cause suicidal behavior in patients with schizophrenia [100]. One of the goals of family interventions is to reduce psychological distress among family members and to decrease expressed emotion in families of patients with schizophrenia. Hence, it is essential to help families of persons with schizophrenia and to explain to patients’ families that their attitude towards the patient can aid or impede recovery. It is necessary to educate families that they need to assist mental health providers to make sure that pharmacological and non-pharmacological treatments are being followed, especially after discharge from inpatient hospitalization. Families of individuals with schizophrenia need to be informed regarding manifestations of suicidality and what needs to be done if an individual with schizophrenia develops suicidal ideation, intent, or plan.

In summary, the only reliable protective factor for suicide in patients with schizophrenia is provision of and compliance with comprehensive treatment. Prevention of suicidal behavior in schizophrenia should include recognizing patients at risk, delivering the best possible therapy for psychotic symptoms, and managing comorbid depression and substance misuse. It is imperative to educate mental health and non-mental health providers about suicide prevention strategies.
